# A Better Quality of Life in Hemodialysis Patients With Viral Hepatitis: Is it a Reality?

**DOI:** 10.5812/hepatmon.15525

**Published:** 2013-11-30

**Authors:** Eghlim Nemati, Mohsen Motalebi

**Affiliations:** 1 Nephrology and Urology Research Center, Baqiyatallah University of Medical Sciences, Tehran, IR Iran

**Keywords:** Quality of Life, Hepatitis, Renal Dialysis

*Dear Editor,*

It is with great interest that we recently read the article by Rostami et al. titled “Health Related Quality of Life in Iranian Hemodialysis Patients With Viral Hepatitis: Changing Epidemiology” (1) published in your valuable journal. This cross sectional study on 4101 hemodialysis (HD) patients focused its message on, and drew attention to, whether chronic viral hepatitis has impact on health related quality of life (HRQoL) when compared to non-infected HD patients (1). 

However, there are few studies about this important issue it is excellent that Rostami et al. worked about this important topic. They showed that not only general health and physical activity were preserved but also health perception may be better among HD patients with viral hepatitis (1), while other studies (2, 3) have shown lower HRQoL in HD patients with chronic hepatitis. It seems that is due to several reasons:

First) it is important to note that in the current study (1), treatment of the patients with viral hepatitis has not been determined because it is known that interferons (IFN) have several adverse effects (4) that can affect quality of life (QoL) during the treatment (5). Akyuz et al. performed a study after IFN treatment to evaluate real QoL and revealed that general health and physical activity which were poor and anxiety were higher in the IFN treated patients as compared with untreated patients (5). This difference may be due to few patients’ treatment with INF in the study of Rostami et al. (1).

Second) a number of studies have shown that many confounder factors such as age, educational level, gender, marital state, sex function, work status and other socioeconomic factors can influence HRQoL (6, 7). It is interest that some of these factors are better in chronic hepatitis patients in the study of rostami and colleagues (1), so better QoL in these patients with chronic hepatitis was predictable.

Third) in a previous study, Bianchi and coworkers (8) evaluated HRQoL in HD patients with chronic hepatitis by comparing two different questionnaires: Nottingham health profile questionnaire (NHPq) that assess distress in six domains (sleep, energy, pain, mobility, emotional reaction and social isolation) with SF-36, a general questionnaire for measuring function and well-being, with eight domains. This study showed that NHPq detected differences in energy, mobility and pain, which were better in treated patients but SF-36 questionnaire did not detect any differences in relation to treatment (8). So, maybe applying different questionnaires in rostami et al. study can help more.

Finally, although this was a great study about HRQoL in a huge number of HD patients with chronic hepatitis, it still requires more studies to evaluate different factors that can affect on HRQoL.
